# The Final Frontier of pH and the Undiscovered Country Beyond

**DOI:** 10.1371/journal.pone.0045832

**Published:** 2012-09-25

**Authors:** Wojciech Bal, Ewa Kurowska, Wolfgang Maret

**Affiliations:** 1 Institute of Biochemistry and Biophysics, Polish Academy of Sciences, Warsaw, Poland; 2 King’s College London, Diabetes and Nutritional Sciences Division, London, United Kingdom; Université Joseph Fourier, France

## Abstract

The comparison of volumes of cells and subcellular structures with the pH values reported for them leads to a conflict with the definition of the pH scale. The pH scale is based on the ionic product of water, *K*_w_ = [H^+^]×[OH^−^].We used *K*_w_ [in a reversed way] to calculate the number of undissociated H_2_O molecules required by this equilibrium constant to yield at least one of its daughter ions, H^+^ or OH^−^ at a given pH. In this way we obtained a formula that relates pH to the minimal volume V_pH_ required to provide a physical meaning to *K*_w_, 
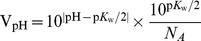
 (where *N*_A_ is Avogadro’s number). For example, at pH 7 (neutral at 25°C) V_pH_ = 16.6 aL. Any deviation from neutral pH results in a larger V_pH_ value. Our results indicate that many subcellular structures, including coated vesicles and lysosomes, are too small to contain free H^+^ ions at equilibrium, thus the definition of pH based on *K*_w_ is no longer valid. Larger subcellular structures, such as mitochondria, apparently contain only a few free H^+^ ions. These results indicate that pH fails to describe intracellular conditions, and that water appears to be dissociated too weakly to provide free H^+^ ions as a general source for biochemical reactions. Consequences of this finding are discussed.

## Introduction

Cells are small. A typical mammalian cell, such as a Chinese Hamster Ovary cell, CHO, has an internal volume of ca. 1.2 pL, of which about 70% is H_2_O [1]. This corresponds to *ca.* 2.8×10^13^ water molecules inside the cell, calculated by applying Avogadro’s number of 6.02×10^23^ molecules per mole to the molar concentration of water *in water*, i.e. 55.56 M. Subcellular compartments are several orders of magnitude smaller than cells. Mitochondria have volumes of *ca.*1 fL, (90% in mitochondrial matrix, and 10% in the intermembrane space), lysosomes of about 30 aL, and the smallest of them, coated vesicles, of about 30 to 800 zL (10^−21^ L) [2]–[4]. In addition, some organelles, such as mitochondria, have variable sizes. They can fuse, divide, and therefore their sizes vary in the range of about an order of magnitude. Thus, in a coated vesicle we find from 7×10^5^ to 2×10^7^ water molecules, in a lysosome 7×10^8^, and in an average mitochondrion 2.3×10^10^

In these organelles, pH values of 6.8 for the mitochondrial intermembrane space, 8.0–8.1 for the matrix, 4–5 for lysosomes, and 5–7 for coated vesicles have been reported [5]. These values have been accepted in the biological community and are hardly ever disputed. In this article, we examine the physical meaning of these pH values

## Results

The pH scale is based on the thermodynamic equilibrium of the water dissociation process, in simplest terms: H_2_O ? H^+^ + OH^−^, with a dissociation constant given by Eq. 1.
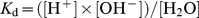
(1)Pure water is dissociated to a very low extent, making the concentration of water molecules in water, [H_2_O], practically constant with respect to the concentrations of its daughter ions. Therefore, the numerator of Eq. 1, the ionic product of water *K*_w_, is also a constant at a given temperature.



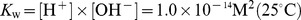
(2a)

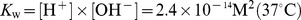
(2b)–[Eq. 2a is the origin of the standard pH scale, with pH defined as log H^+^] [6]. Importantly, *K*_w_ is a very small number, which means that there are a myriad of undissociated water molecules per one water dissociated into H^+^ and OH^−^– ions. Examples 14 show how many water molecules are undissociated for several values of pH and temperature. In these examples, we first defined conditions of temperature and pH to determine molar concentrations of H^+^ and OH^−^ ions using the appropriate *K*_w_ value of Eq. 2a or 2b. Then we selected the lower of these values and calculated the number of H_2_O molecules per one H^+^or OH^−^[ ion, using the H_2_]’O value. Finally, we applied Avogadros number, *N*_A_, to find the volume of these water molecules.

**Example 1:**° pH 7.0 and 25C

[H^+^] = 10^−7.0^[ M, thus OH^−^] = 10^−7.0^ M

1 H^+^× per 5.5610^8^ water molecules

V_pH_ = ×1.6610^−17^ L (16.6 aL).

**Example 2:**° pH 6.8 and 37C

[H^+^] = 10^−6.8^[ M, thus OH^−^] = 10^−6.8^ M

1 H^+^× per 3.5910^8^ water molecules

V_pH_ = ×1.0710^−17^ L (10.7 aL).

**Example 3:**° pH 7.4 and 37C

[H^+^] = 10^−7.4^[ M, thus OH^−^] = 10^−6.2^ M,

1 H^+^× per 1.410^9^ water molecules

V_pH_ = ×4.210^−17^ L (42 aL).

**Example 4:**° pH 5.0 and 37C

[H^+^] = 10^−5^[ M, thus OH^−^] = 10^−8.6^ M

1 OH^−^× per 2.2210^10^ water molecules

V_pH_ = ×710^−16^ =  L (700 aL0.7 fL).

×Example 1 shows that it takes as much as 16.6 aL of pure water at neutral pH and as many as 5.5610^8^ of mutually interacting water molecules to maintain a single H^+^ and a single OH^−^!! ion This critical result provides the absolutely minimal volume for defining pH 7, simply because there cannot be less than one molecule dissociated at a given moment We shall label this volume as V_pH_°. Water is more prone to dissociate at higher temperatures, resulting in a smaller minimal volume for neutral water solution at 37C (Example 2), but the difference is relatively small. Moreover, any deviation from neutral pH, up or down (Examples 3 and 4), requires a *larger*[ number of water molecules to define pH. This is because the concentrations of protons and hydroxyl ions, H^+^][ and OH^−^], are coupled by *K*_w_[. An increase of H^+^][ is accompanied by a decrease of OH^−^] and *vice versa.* Generally, V_pH_, the minimal volume required to maintain at least one H^+^ or OH^−^ ion at a given pH, *indeed the final frontier of pH*, is given by Eq. 3a:
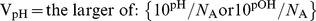
(3a)


Eq. 3a can be rearranged into an alternative form (Eq. 3b). The expression 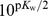
is the concentration of H^+^ and OH^−^ ions in a neutral solution at a given temperature.
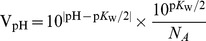
(3b)


**Figure 1:**
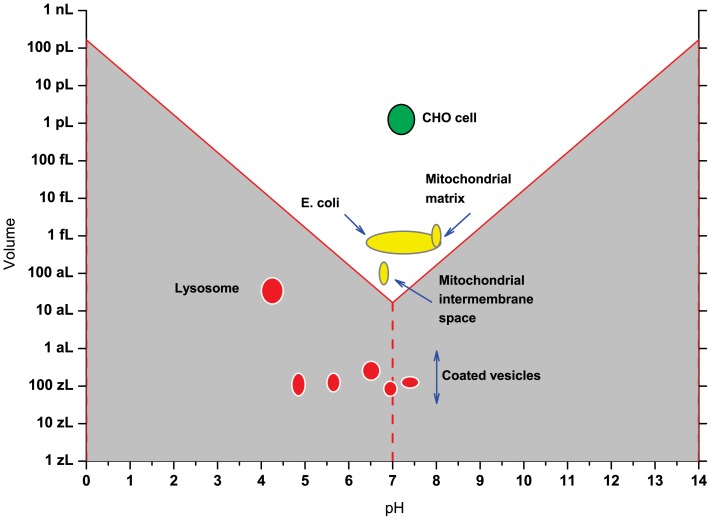
The volumes and reported pH values of biological structures [2]–[5], [7], compared with V_pH_ as defined by Eq. 3b.

In Fig. 1, the volumes of biological structures are juxtaposed with V_pH_. Clearly, the smallest of subcellular structures listed above, coated vesicles, are far below this limit, regardless of the experimental pH value that has been inferred. Lysosomes are larger, but they are also below the limit, because of their reported acidic pH. Other important structures and organisms linger just above the limit. For mitochondria, the combined volume and pH data indicate that there are just a few H^+^ ions (n_H_) in the matrix and the intermembrane space (Examples 5 and 6). Similarly sized bacteria also would appear to support their metabolism on only a few H^+^ ions [7].

**Example 5:** =  pH 6.8, V0.1 fL (mitochondrial intermembrane space)

[H^+^] = 10^−6.8^ = × M, V710^−17^ L

n_H_ = ×6.0210^23^×10^−6.8^××710^−17^

n_H_ = 6.7

**Example 6:** =  pH 8.05, V0.9 fL (mitochondrial matrix)

[H^+^] = 10^−8.05^ = × M, V6.310^−16^ L

n_H_ = ×6.0210^23^×10^−8.05^××6.310^−16^

n_H_ = 3.4

“”“”““The above facts reveal two major issues related to cellular pH, which we may call the hard and the soft pH crisis. The hard pH crisis regards the grey zone in Fig. 1“”“”“”, in which the pH definition cannot be fulfilled (or simpler: where pH is not defined). One can argue that it is possible to consider concentrations lower than one molecule by using time averages of the incidence of having asingle molecule in a given volume. But, in fact we obtain a system that oscillates between the pH and no pH states, with no pH times increasing with the distance from the limit. As a result, the V_pH_ frontier becomes somewhat blurred, but the problem remains. Our analysis is still valid if we substitute concentrations in Eq. 2 with activities, because activity coefficients are never higher than 1. As a result, the V_pH_ obtained using activities is even higher, aggravating rather than solving the problem.

“”The soft pH crisis is equally bothersome. Let us consider a mitochondrion, an organelle with a femtomolar volume generating chemical energy for the metabolism of eukaryotic cells [5], [8]. This energy is generated by the H^+^ gradient across the phospholipid bilayer surrounding the mitochondrial matrix (into the matrix). The synthesis of one ATP molecule is accompanied by the transfer of up to three H^+^ ions along the gradient, from the intermembrane space to the matrix. Thousands of ATP synthase complexes are considered to be simultaneously active [8]. Yet, as shown in Example 5, simple and obvious calculations demonstrate that there may be no more than *seven* H^+^! ions in total in the whole intermembrane space

## Discussion

How is it possible then that H^+^ and OH^−^ dependent biochemical reactions occur even in the smallest cellular compartments (e.g. proteolysis in lysosomes) and that pH values are reported for them, when in fact there are only a few or no free H^+^ ions present, and hence *no*? pH And how is it possible that myriads of acid/base reactions occur simultaneously in cells, where H^+^? ions are still so few that they are, in principle, countable (Example 7)

**Example 7:** =  pH 7.2, V0.6 pL (water contents of the CHOcell cytosol)

[H^+^] = 10^−7.2^ = × M, V610^−13^ L

n_H_ = ×6.0210^23^×10^−7.2^××610^−13^

n_H_ = 22790

“”The issue of pH measured in the no-pH zone of Fig. 1 can be partially explained by the fact that molecular probes used for such measurements in fact report their own protonation state and not the concentration of free protons in solution. This can be depicted by the following thought experiment. Let us introduce into a lysosome 100 molecules of a pH sensitive fluorescent sensor SH, whose p*K* (5.0) is the same as the pH in this structure, according to recommended procedures [9]. Let us then assume that these molecules get into the lysosome via the cytosol, where they assume the protonation pattern of corresponding to the cytosolic pH 7.2. At this pH all sensor molecules will be in the deprotonated form S^−^, but to report pH 5 from the lysosome 50 of them will have to become protonated into SH. But, as we demonstrated above, the number of H_2_O molecules inside the lysosome allows for only 2 or 3 free H^+^ ions. Obviously, there must be another source of protons for the sensor to report pH 5.

Let us consider this point more closely. The re-protonation of a sensor in the thought experiment depends on an interaction with a proton-donating molecule. Thus the state of the sensor will depend on the equilibrium constant of the direct interaction of the donor/acceptor type, and thus the sensor does not measure pH, but the equilibrium of this interaction. These considerations are valid not just for sensors, but for all acid/base reactions in the cellular milieu. As a result we paradoxically come up with a pH independent (and water dissociation independent) acid-base chemistry that occurs by many pairwise direct proton and hydroxide exchange reactions between molecules.

!Another important point to make in this context is that in order to make valid observations the sensor should not affect the equilibrium condition, and thus there should be a significant excess of the analyte (protons in this case) over the probe. The condition is not met for subcellular compartments The assumed equivalence of the protonation state of the fluorophore and pH is a fallacy as it is based on calibration experiments performed in macroscopic, mL volumes in test tubes, where, unlike at the nanoscale, H^+^ and OH^−^ ions are in abundance [9]. In this way, the *availability* of H^+^ ions to protonate the probe is confused with the concentration of *free* H^+^ ions in the solution.

“”–This interpretation leads us to one possible solution of the soft pH crisis. Calculations such as those provided in Examples 57 indicate that water is too weakly dissociated to be a direct donor or acceptor of H^+^ and OH^−^ ions at very low biological volumes, even if one can still define the pH formally. It appears that H^+^ ions can be shuttled to reaction centers by other biomolecules, whose concentrations are not constrained by Eq. 2. Best candidates for such molecules are those having pK values close to 7, and are thus capable to serve as donors and acceptors of H^+^ ions. One obvious group of candidates for such proton donors, available in millimolar and higher concentrations in every intracellular locale are phosphates, e.g. inorganic phosphate, nucleotides and membrane phospholipids. If we consider that radius of a typical lysosome is 180 nm []3×, the area of the inner face of its phospholipid membrane will be approx. 410^4^ nm^2^Å. Given that one phospholipid head covers an area of about 60^2^%× and about 50 of the membrane surface is occupied by proteins, we have roughly 710^5^ phospholipid heads in a lysosome [10], [11]%. About 50 of phospholipids carry amine groups with a p*K*!– of about 9, indicating that under cellular conditions most of them will be protonated. At this point we end up with a reservoir of over three hundred thousand protons in only just one lysosome Interestingly, the pK value of the phospholipid amine can be lowered by 12 log units, depending on the phospholipid composition of the membrane []12’. Thus phospholipid bilayers may be a significant and tunable source of protons for cellular reactions. It may also the very reason (reason detre) of why reactions occur on cell membranes.

[]The general view that emerges from the above thought experiments and calculations is that the intracellular pH is in fact a composite of many pairwise proton exchange interactions between individual protonable molecules. Chemical pH sensors are such molecules. As a consequence, the pH reported for the same compartment by chemically different sensors may be different, and the same sensor may report different pH values, depending on the chemical composition of the compartment.

An alternative possibility, though appearing to us as less likely, but one that should not be dismissed *a priori*, is that at the nanoscale *K*_w_ is no longer a correct thermodynamic constant (in other words H^+^ and OH^−^ ions are no longer in equilibrium with H_2_O). New experiments, performed under appropriate volume constraints, will be needed to explore this emerging *Undiscovered Country* of cellular acid/base chemistry. Whatever their outcome will be, when studying and discussing the intracellular pH one always has to bear in mind that the pH definition is *solely* based on the water dissociation process and it cannot be considered without reference to this fact.

### Conclusions

In this work, we started from the textbook definition of pH and well known volumes of cells and their compartments to demonstrate that (a) the definition of pH is not fulfilled for biologically relevant attoliter volumes, and (b) water is not sufficiently dissociated to provide free H^+^ ions for biochemical reactions. We consider this obvious discrepancy to be of a high importance for molecular mechanisms of cellular processes and hope that it may raise more interest in this fundamental and complex issue of the quintessential biological unit: the cell.
